# ‘It was just the given thing to do’: exploring enablers for high childhood vaccination uptake in East London’s Bangladeshi community—a qualitative study

**DOI:** 10.1136/bmjph-2024-001004

**Published:** 2025-01-16

**Authors:** Ifra Ali, Sadie Bell, Sandra Mounier-Jack

**Affiliations:** 1 Global Health and Development, London School of Hygiene & Tropical Medicine Faculty of Public Health and Policy, London, UK; 2 London School of Hygiene & Tropical Medicine, London, UK

**Keywords:** Public Health, Primary Prevention, Communicable Disease Control, Public Health Practice, Vaccination

## Abstract

**Background:**

Despite being an underserved ethnic minority group, characteristics which have been associated with low vaccine uptake, the Bangladeshi community in the UK exhibits high childhood vaccination uptake for several vaccines, including measles, mumps and rubella compared with several ethnic groups. This study explored key enablers for early childhood vaccination uptake among the Bangladeshi community in East London, UK.

**Methods:**

A qualitative study using semi-structured interviews was conducted with 23 Bangladeshi parents 11 primary healthcare professionals (HCPs) and 5 community service providers (CSPs) involved in delivering childhood vaccination services, predominantly in the East London borough of Tower Hamlets. Parents were recruited purposively from the community while HCPs and CSPs were recruited from relevant organisations. The Social Ecological Model (SEM) was used as a theoretical framework for data collection and analysis. Data were analysed thematically.

**Results:**

Multilevel vaccination enablers identified by parents, HCPs and CSPs across the different levels of the SEM included: parental trust in the immunisation programme and HCPs; a rigorous call and recall service; the normalisation of receiving childhood vaccinations within the Bangladeshi community and the availability of culturally tailored and accessible vaccination services.

**Conclusions:**

This study highlights how multi-level trust in a vaccination programme can propel positive vaccine uptake in an underserved, ethnic minority population. Our findings suggest culturally sensitive, person-centred delivery of vaccination services, alongside leveraging community dynamics and trusted social networks, are imperative to meeting the informational, linguistic and cultural needs that facilitate vaccine uptake within the Bangaldeshi community. We recommend using existing trusted community networks to disseminate tailored vaccine information and actively reminding parents about due vaccinations to promote uptake amongst other underserved, ethnic minority communities with low uptake in high-income settings. Further research involving non-immunising parents is recommended to gain more comprehensive insight into vaccine decision-making within this community.

**WHAT IS ALREADY KNOWN ON THIS TOPIC**

- In the UK, early childhood vaccination uptake is often lower among underserved populations, including ethnic minority groups. However, the underserved Bangladeshi community in the UK has exhibited high childhood vaccination uptake. Factors underpinning this uptake have not been explored.

**WHAT THIS STUDY ADDS**

- Multi-level enablers to childhood vaccination uptake were identified across each level of the Social Ecological Model for an underserved, ethnic minority community which have not previously been explored. The enablers include trust in vaccinations and healthcare professionals, positive community dialogue on vaccinations, the delivery of culturally tailored, accessible vaccination services and consistent call and recall efforts.

**HOW THIS STUDY MIGHT AFFECT RESEARCH, PRACTICE OR POLICY**

- Our findings on the value of building multilevel trust, providing culturally tailored vaccination services, propagating positive community-level messages, implementing a rigorous call and recall programme and issuing vaccination records may inform the adaption of vaccination services for underserved populations. These strategies could improve vaccine uptake among culturally diverse populations with low uptake in other high-income settings.

## Introduction

 Childhood vaccinations are an efficacious, preventative method of reducing the burden of infectious diseases and associated mortality^1 2^ which are routinely offered to children by the National Health Service (NHS) in the UK^3^ (see table 1 for schedule). Despite the established success of childhood vaccinations, avoidable inequalities in uptake exist within and between populations.^4 5^ Specifically, lower uptake has been observed among socially disadvantaged groups, including ethnic minority populations such as those from South Asian, Black African and Black Caribbean backgrounds, individuals of lower socioeconomic status and migrants in the UK.^6^,^8^ Distrust in healthcare systems and vaccinations, low health literacy and difficulties accessing healthcare services are notable barriers to vaccination experienced by such groups in high-income settings.^9 10^ Nevertheless, the observed relationship between ethnicity, socioeconomic status and vaccine uptake is complex and not always linear.^11 12^

**Table 1:** UK vaccination schedule for babies and children^61^

Age	Vaccines
8 weeks	6-in-1 vaccineRotavirus vaccineMenB
12 weeks	6-in-1 vaccine (2nd dose)Pneumococcal (PCV) vaccineRotavirus vaccine (2nd dose)
16 weeks	6-in-1 vaccine (3rd dose)Men B (2nd dose)
1 year	Hib/MenC (1st dose)MMR (1st dose)Pneumococcal (PCV) vaccine (2nd dose)MenB (3rd dose)
2–10 years*	Influenza vaccine (given annually)
3 years and 4 months	MMR (2nd dose)4-in-1 preschool booster
12–13 years	HPV vaccine
14 years	3-in-1 teenage boosterMenACWY

* Upper age limit may vary.

One key example is the underserved, ethnic minority Bangladeshi community in the UK. During the 1970s, large numbers of Bangladeshis primarily from the Sylhet region of Bangladesh (Northeastern Bangladesh) migrated to England in pursuit of economic opportunities.^13^ According to latest estimates, Bangladeshis comprise 1.1% of the total population in England and Wales,^14^ of which approximately 52% were born in the UK and 48% in Bangladesh.^15^ The highly socioeconomically deprived East London borough of Tower Hamlets hosts the largest Bangladeshi population in England and Wales, where they constitute 34.6% of the borough’s population.^16^ The neighbouring borough of Newham also hosts a relatively large proportion of Bangladeshis (approximately 16%).^16^ According to recent data, early childhood vaccination coverage in the East London boroughs of Tower Hamlets and Newham is lower than the national average^17^

The Bangladeshi population is one of the most disadvantaged, growing ethnic minority groups within the UK as measured by a range of indicators such as income and housing.^18^ Bangladeshis are approximately two times as likely as white people in the UK to be living on a low income.^18^ They also experience poorer health compared to several other ethnic groups^19^ including higher rates of heart disease-related mortality^20^ and have reported difficulties in accessing healthcare.^21^

Despite these characteristics, studies have observed higher childhood vaccination uptake among Bangladeshi communities compared with other ethnic groups in the UK.^5^ Recent research found Bangladeshi children were two times as likely to have received at least one of the two recommended doses of measles, mumps and rubella (MMR) vaccination compared with white groups in the UK.^22^ Similarly, in North-West London, the MMR vaccine (first dose) uptake was 84.6% among the Bangladeshi community, higher than the Afro-Caribbean (74.7%) and white group (57.5%).^23^ Higher uptake of the Rotavirus vaccine^24^ and the third dose of diphtheria, tetanus toxoid and pertussis (DTP3)^5^ has also been observed among Bangladeshi groups relative to other ethnic groups. Similarly, high uptake of the MMR (95%) vaccine was reported in Bangladeshi infants in Manchester compared with white British infants (88%).^25^

It is vital to understand the enablers driving high childhood vaccination uptake within the Bangladeshi community, and to our knowledge, no previous research has investigated this. This research may generate learning that can inform strategies for improving vaccination uptake among other underserved populations with low uptake.

## Methods

### Theoretical framework

**Figure 1:**
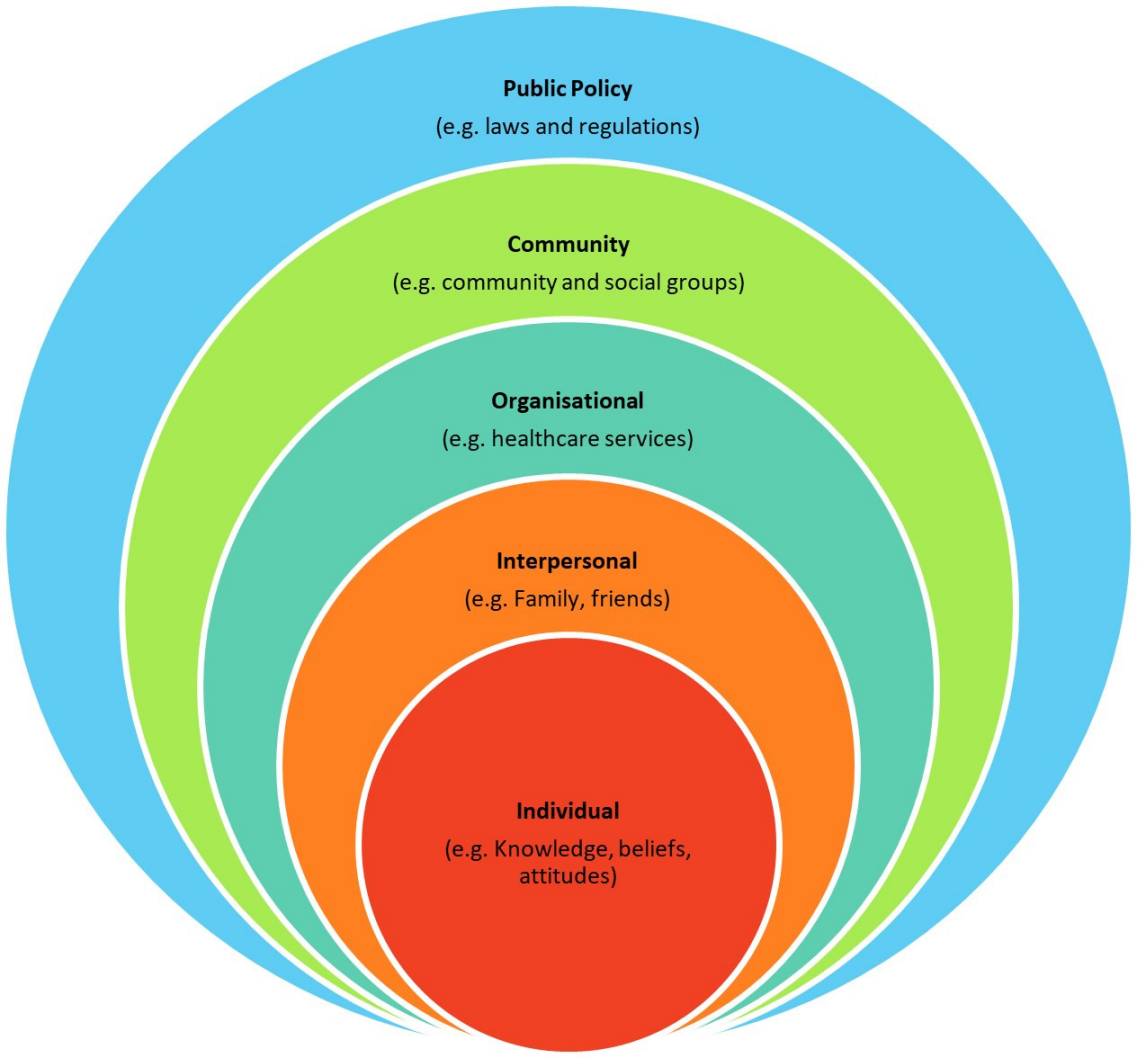
The Social Ecological Model.^26^

This qualitative study used the Social Ecological Model (SEM) (see figure 1) as a theoretical framework to guide data collection and analysis The SEM acknowledges the multi-level influences on vaccination uptake at the individual, interpersonal, organisational, community and policy level.^26^ The SEM has previously been employed to understand vaccination behaviours in various populations.^27 28^ During data collection, we used the SEM to guide the domain of the questions in our topic guides. During analysis, the SEM informed the categorisation of coding, interpretation and presentation of our themes.

### Recruitment strategy

A purposive sampling method was used to recruit Bangladeshi parents of children aged 0–4 years residing within Tower Hamlets or Newham. This age criterion was chosen to correspond with the UK early childhood immunisation schedule, while these boroughs were selected for recruitment as they house large Bangladeshi populations. Culturally tailored posters were designed and distributed in person in community settings, including children and family centres, libraries and mosques. Community-based organisations that serve Bangladeshis in East London also shared the poster via their social media platforms.

Second, healthcare professionals (HCPs) and community service providers (CSPs) involved in the delivery of childhood vaccinations across Tower Hamlets and Newham were recruited to obtain insight into the service delivery factors that may be influencing uptake. CSPs included local authority (Tower Hamlets council) members involved in organising the local delivery of the childhood immunisation programme, alongside members of voluntary organisations involved in promoting vaccination awareness within the community. HCPs were recruited from GP practices across Tower Hamlets and Newham, with support from the NIHR East London Clinical Research Network.

### Data collection

One-to-one semi-structured interviews were conducted with parents, HCPs and CSPs by IA between March and May 2022. An outsourced Bangladeshi interpreter facilitated interviews with parents who had an English language barrier, verbally translating information and questions into Bengali or Sylheti (a commonly spoken dialect). The interpreter was briefed on following a standardised protocol for interviews to minimise subjectivity. Informed consent was either obtained verbally, and audio recorded or electronically emailed to the researcher before participation. Interviews lasted between 30 and 40 min and were conducted via telephone or virtually using video conferencing software per participant preference and audio recorded. Parents received a £15 voucher as reimbursement for their time. Data collection was ceased at the perceived point of data saturation, identified by the absence of new themes emerging from the data analysis.^29^

Separate topic guides were developed for parent (see online supplemental material 1) and HCPs/CSPs interviews (see online supplemental material 2). Questions were informed by the SEM, existing literature^28 30^ and explored uptake of vaccinations, knowledge, perceptions and experiences of receiving and delivering childhood vaccinations with this community group.

### Public involvement

One-to-one discussions took place with three Bangladeshi community members to develop the study documents and recruitment strategy. Input was obtained on the information sheet, pretesting the topic guide, recruitment poster and recruitment strategy. Materials including the poster and topic guide were adapted using imagery and wording to increase cultural sensitivity, and a culturally informed recruitment strategy was devised.

### Data analysis

Interviews were pseudonymised and transcribed verbatim by a third-party transcription service. For interviews conducted in Bengali/Sylheti, the interpreter verbally conveyed the participants’ responses in English, so translation services were not required.

**Figure 2:**
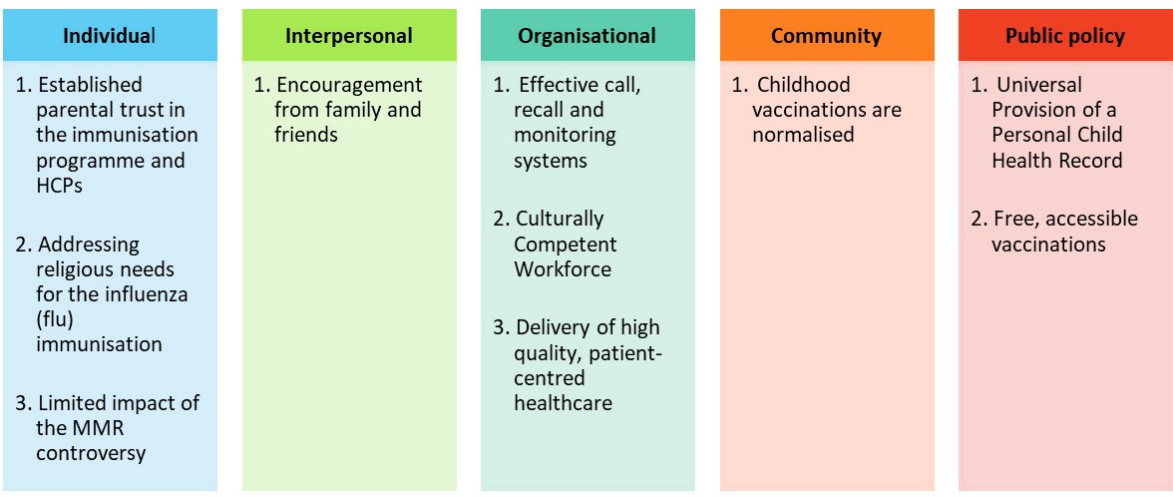
Thematic results corresponding to each level of the Social Ecological Model. HCPs, healthcare professionals; MMR, measles, mumps and rubella.

Using NVivo, an abductive thematic analysis following existing guidance was conducted, which consists of a combined inductive and deductive approach to analysis.^31 32^ The initial, descriptive coding of transcripts was completed by IA and refined through regular, detailed discussions with SM-J to enhance the rigour of the analysis and achieve consensus. Codes were analysed semantically and grouped together into categories, under each level of the SEM. These categories were further developed into themes, which are summarised in figure 2.

## Results

### Participant characteristics

23 Bangladeshi parents, 11 HCPs and 5 CSPs participated. No participants withdrew from the study.

Most parents were mothers (n=19). The age range of parents disclosing this information was between 27 and 42 years. Almost all parents (n=21) were residents of Tower Hamlets except for one who lived in Newham and one unspecified. Nine parents were born in the UK while the remaining 14 had been settled in the UK for a minimum of 3 years. Most parents self-reported completing the immunisation schedule (to date) for their child(ren), with only one failing to recall this information and one child having received all except the influenza vaccination. Parent characteristics are detailed in online supplemental material 3.

The 11 HCPs interviewed included a healthcare assistant (n=1), practice nurses (n=2), paediatric nurse (n=1), lead nurse (n=1), lead nurse manager (n=1), GPs (n=4) and an emergency medicine doctor (n=1), all of whom were based within Tower Hamlets or Newham. The CSPs interviewed included public health programme managers (n=3), a councillor (n=1) and a senior member of a local community organisation (n=1), all of whom were based within Tower Hamlets.

### Thematic results

#### Individual-level

##### Established parental trust in the immunisation programme and HCPs

Most parents expressed a high level of trust in the safety and importance of the childhood immunisation programme. Vaccinations were considered essential to protecting their child(ren) from future illnesses and this trust was attributed to having observed the value and safety of the vaccines across generations.

Because you know [the] childhood one is an old vaccine and everyone has it, and we know it’s not big side-effect or anything. (Parent 11, age 30–39)

High trust in HCPs was also pivotal in parental decision-making, with many parents expressing strong confidence in the advice given by HCPs. GPs were generally held in high esteem due to their perceived authoritative status, a social hierarchy which encouraged ‘blind compliance’ among some parents. Parents and HCPs stated this hierarchical perception was common among migrant, first-generation Bangladeshi parents, who often exhibit lower health literacy and agency in their health decisions. These parents also reportedly had limited understanding of the various vaccinations offered but were inclined to vaccinate due to their trust in the HCPs and vaccines. Contrastingly, second-generation parents demonstrated a higher level of knowledge regarding the vaccination programme and more confidence in independently seeking out relevant vaccination information.

I don’t know how to explain this, but I find that a lot of Bangladeshi parents that I come in contact with, they kind of think, ‘Okay, if I listen to the health professional, nothing can go bad’, it’s that attitude where they know best, you should just listen to them. (HCP 11, Paediatric Nurse)

##### Addressing religious needs for influenza (influenza) immunisation

Several HCPs had observed reluctance and refusal from some Bangladeshi parents in undergoing the (nasal) influenza immunisation due to it containing Porcine Gelatine, an ingredient regarded as impermissible (beyond medical exemptions) for consumption in the Islamic faith (the predominant faith of this population). Nevertheless, only one parent expressed hesitancy to undergo the influenza immunisation for this reason. Some HCPs and CSPs emphasised raising awareness about the permissible (*halal*) alternative form of influenza immunisation (non-porcine-containing vaccine) significantly facilitated uptake, although limited stock of this alternative in practices can result in delayed immunisation. Additionally, some HCPs stated that the overarching medicinal value of the porcine-containing immunisation has been deemed permissible by some Muslim scholars, and communicating this scholarly view with parents can encourage uptake.

Overall, parents, HCPs and CSPs iterated that transparency regarding the vaccination ingredients and provision of information regarding suitable alternatives was the most effective means of navigating religious concerns surrounding the influenza immunisation and encouraging uptake.

I didn’t used to take the gelatine one, but one time, maybe last year, when I explained that I didn’t want to give them, they gave me the other one and said it was a flu vaccine without gelatine, there’s a halal one. So, if I want to, I can take it. So, this is the first time I heard there is a halal one. (Parent 10, age 30–39)

##### Limited impact of the MMR controversy

Only four parents expressed awareness of the claimed association between the MMR vaccine and autism,^33^ most being second or third generation Bangladeshis. Of these parents, all except one stated they would still accept the MMR vaccine as they deemed the claim invalid. One parent stated they remained apprehensive and would likely delay the vaccination until their child was older. Nevertheless, all parents reported their child(ren) had received the MMR vaccination where eligible with most viewing it no differently to other recommended vaccines.

Consistently, some HCPs observed hesitancy with the MMR vaccine specifically among second and third-generation Bangladeshi parents. This observation was attributed to these parents having increased agency in their health decisions and higher exposure to conflicting information online surrounding the MMR vaccine.

I would say any parents under the age of 40, maybe between 20 to 30 are quite reluctant on having vaccinations because they would like to do their own research about MMR. It’s all about what’s on the news, and what’s on the media, whereas people, mothers and fathers over the age of 40, if you just try and explain it to them and give them the leaflet, I think that they’re quite happy to go ahead with the vaccine, especially with the MMR. (HCP 7, Healthcare Assistant)

### Interpersonal level

#### Encouragement from family and friends

Most parents stated their family and friends held a positive view of childhood vaccinations and encouraged them to vaccinate their children. Similarly, some HCPs reported encouragement from family members was a prominent enabler of vaccination within this tight-knit, family-orientated community. Several parents advocated their family members with children were a reliable, and commonly referred to information source for childhood vaccines. A few stated English-speaking relatives were often called on to translate vaccination information for non-English-speaking parents.

Because I think, you know, this is my first baby, so naturally I’m going to seek advice from my friends and family because they’ve been through it, they’ve gone through it, they know what they’re doing. So, it definitely influences my decision. How much of that I’d take it into account would probably be dependent on the person that I listen to or the person who’s telling me or how much experience that they’ve had. (Parent 13, age 30–39)

Counterproductively, the counsel of family and friends appeared to also be a means of propagating vaccination hesitancy. A few parents narrated anecdotes from family and friends of children developing autism and other serious side effects soon after receiving the MMR vaccine. Most of these parents stated such anecdotes made them warier of the MMR vaccine.

For MMR, some people asked me not to do that because they said it might be, you know, they might get side-effects and stuff. (Parent 5, age 30–39)

### Organisational level

#### Effective call, recall and monitoring systems

Regular vaccination and appointment reminders provided by HCPs were vital enablers for most parents. Some, particularly migrant parents with less awareness of the UK health services, stated they would have lacked awareness, possibly delaying or missing their child’s vaccinations without this proactive communication. The process of booking vaccination appointments was also deemed easy and convenient, with only some parents experiencing difficulties in booking the BCG vaccination.

They call me; the easiest thing is they call me. I didn’t have to bother, I didn’t have to be reminded about their vaccine and stuff but they called me to book my next appointment, the time for my children to get their next vaccine. They remember me, I didn’t remember them. (Parent 5, age 30–39)

Consistently, several HCPs and CSPs attested the well-synchronised call and recall system, and opportunistic reminders in GP practices across the region, were fundamental in promoting uptake. They also highlighted having an efficient, user-friendly computer system that flags missed vaccinations and sends reminders to parents was crucial in facilitating the call and recall programme. However, it was acknowledged that high workload and insufficient staffing can hinder the consistency of call and recall efforts.

#### Culturally competent workforce

HCPs and CSPs highlighted most GP practices within Tower Hamlets and Newham have an ethnically diverse workforce that reflects the population they serve. It is thus common for Bangladeshi parents to receive vaccinations for their child from a Bangladeshi nurse or doctor. A few parents, particularly those with an English language barrier, preferred being seen by a Bangladeshi HCP as this commonality facilitated communication and enabled their cultural and religious values to be implicitly understood. For these parents, face-to-face verbal communication regarding vaccinations in Bangla/Sylheti was preferred to translated, written information due to Sylheti being only a verbal language. Generally, most parents felt HCPs delivered culturally competent care, demonstrating an understanding of the cultural values, religious beliefs and communication needs of the Bangladeshi community. Examples of culturally tailored care provisions included accessible interpreters, translated vaccination leaflets, and non-porcine-containing vaccines. From both service user and delivery perspectives, it appeared the diverse needs of Bangladeshi parents were being understood and met.

When I used to speak to the nurses, […]. When I didn’t understand, they used to say, “Do you want an interpreter?” and they would get an interpreter. Sometimes I would have a Bengali doctor, but not always, and then they would get an interpreter to translate. (Parent 20, age 30–39)

#### Delivery of high-quality, patient-centred healthcare

Almost all parents stated they had a positive relationship with their local immunisation provider (GP or nurse). Most felt comfortable asking questions, felt their queries were handled effectively, and experienced patient-centred care when getting their child(ren) vaccinated. As such, GPs were identified as a key point of contact for credible information on childhood vaccinations, particularly for parents with limited English and digital literacy who were less able to independently source information online. Other parents primarily sought advice and support on the immunisation schedule from midwives and health visitors who were key healthcare providers for their child(ren). The convenient location of GP practices within the community was also an important enabler.

They’re so good. I mean, my practice itself is really good anyway but I think they just make an effort with the child, they make—I mean, they were really considerate of my feelings as well. They were like, OK, they can tell I’m very not OK with it so they got my husband to hold the baby and they’d advise us. They were just very chatty, very reassuring, you know, very kind of just give the information that we need, as parents, to kind of make sure that we’re at ease with what we’re about to do to my son. (Parent 13, age 30–39)

### Community level

#### Childhood vaccinations are normalised

Undergoing childhood vaccinations was viewed as a positive, socially normalised health behaviour within the Bangladeshi community. Some parents incorrectly believed childhood vaccinations were a mandatory legal requirement for UK residents. Several parents attested this belief is common within the community, particularly among first-generation parents, and may explain the observed high uptake. Overall, these norms and beliefs meant parents experienced little to no deliberation in their decision and considered it as ‘a given’ that they would vaccinate their child(ren).

It was compulsory because as I said, I’ve had it, my siblings have had it, we’ve been born and brought up in this country so it almost felt like it was just the thing to do that you just, and I think everywhere you go, everybody would ask ‘Have you had your childhood immunisations?’ … So it was quite just the given thing really, that you just have it done. (Parent 15, age 40–49)

Additionally, some parents and HCPs stated positive community dialogue on childhood vaccinations may be underpinning the positive uptake. Bangladeshi parents often share their vaccination experiences within the community, as this is usually positive, it indirectly encourages uptake within the community. Several participants also stated undergoing childhood vaccinations is a routine, culturally accepted healthcare procedure in Bangladesh, which may be reinforcing a continued acceptance among the UK Bangladeshi community.

Another thing is, within the community is, if my child has a vaccine, I’ll talk to my neighbour, my neighbour will talk to someone who’s gone down to the shops, within the mosque, this, that. Wherever, within the community, they are talking to each other, so the conversation on vaccinations is not new to the community. (HCP 9, Lead Nurse)

### Public policy

#### Universal provision of a ‘Personal Child Health Record’

Many parents reported a strong reliance on the Personal Child Health Record (PCHR) (commonly referred to as the ‘red book’) which is routinely issued to parents following their child’s birth. The PCHR served as personalised and practical tool for tracking their child’s immunisations and upcoming appointments, thus facilitating uptake. Consistently, some HCPs expressed the PCHR was beneficial for consistently scheduling vaccination appointments and verifying a child’s vaccination status, especially when parents were unable to recall this information.

I guess, everything’s just always referred back to the red book and I think the red book is probably, you know, the holy grail as people kind of tend to say with different things. So for him and vaccinations, understanding what the vaccinations are, when he needs them, at what age or what month he’ll have them and what they are, I think pretty much the red book is kind of self-sufficient in providing that information for it. (Parent 13, age 30–39)

However, a few HCPs reported challenges in tracking the vaccination records of Bangladeshi families who migrate to Tower Hamlets from Bangladesh or other European countries and do not possess a PCHR. These families are often unable to provide documentation to verify their child’s vaccination history. Where vaccination records are unobtainable at registration with a UK GP practice, the child may be transferred onto the UK schedule and vaccinated with the age-appropriate vaccines or in some cases considered unimmunised and offered a full course of immunisations according to the UK childhood immunisation guidelines. One HCP observed some parents are hesitant to ‘restart the schedule’ as they are wary of their child receiving repeated vaccinations. This hesitancy can sometimes result in missed vaccinations.

So the complexity that I have in my job is … to try track people’s health records when leaving Bangladesh, then moving to Europe, and sometimes it’s several countries in Europe, before they get to the UK, it’s quite difficult, and not many people have accurate records, or remember having those immunisations. (HCP 3, Emergency Medicine Doctor)

#### Free, accessible vaccinations

In the UK, children are offered vaccinations via the NHS, with no direct costs to parents. Some parents recognised this as an enabler. They made comparisons to Bangladesh, where vaccinations are not always free, nor accessible, a contrast which increased their willingness to use the UK’s free vaccination programme. Importantly, one CSP highlighted the importance of reducing indirect costs to parents incurred because of travelling to clinics and arranging childcare to sustain this positive uptake.

I think, I mean, obviously it might sound wrong and might be without substance, but I think because compared to Bangladesh where you have to pay for your medication and treatment, I think it’s just that pleasure to rely on that it’s being free, and that support is being issued with a cost of a single pence is what drives more parents and adults to get their children vaccinated because they know it’s not going to have any implication on them financially. (Parent 21)

## Discussion

This study highlighted several, multi-level enablers to receiving childhood vaccinations among East London’s Bangladeshi community. While these enablers are presented under specific levels of the SEM, it is key to note these levels are interconnected, thus the identified themes may span across multiple levels. Key enablers included parental trust in childhood vaccinations and HCPs, culturally tailored and patient-centred healthcare and resources, effective call and recall efforts, a community-wide acceptance of the vaccination programme, and universal provision of free vaccinations and records. Almost all parents reported their child(ren) had completed the recommended vaccination schedule to date, supporting previous findings of high uptake among Bangladeshi parents.^5 25^

Parental acceptance and trust in a childhood vaccination programme are core predictors of uptake.^34^ Bangladeshi parents exhibited multilayered trust in the safety and importance of childhood vaccines, interpersonal trust in the vaccination advice imparted within their social network, alongside institutional-level trust in HCPs and the UK healthcare system, all of which facilitated their uptake of childhood vaccines. This contrasts with the high levels of distrust in vaccination programmes and healthcare providers which has been documented among several ethnic minority populations including Black African and Black Caribbean and South Asian communities within the UK.^35 36^ Reasons underpinning this distrust are multifold and include significant historical medical injustices that have afflicted these communities and language barriers which impede access to correct, comprehendible vaccine information.^35^

The atypical high trust observed within the Bangladeshi community was cultivated by two key experiences. First, parents felt the vaccination programme was delivered in a culturally attuned manner, meeting their informational, linguistic and religious needs. Providing culturally tailored healthcare and offering diverse communication channels are known enablers to engaging ethnic minority patients.^37^,^39^ Second, most parents affirmed the positive rhetoric about the importance and safety of childhood vaccinations within the cohesive community reinforced their trust in the programme. Evidently, positive community dialogue on vaccinations with trusted community members can promote vaccine uptake.^40^

Our findings diverge from those reported in studies of Bangladeshi families in other high-income settings, particularly among recent migrants. For example, research including Bangladeshi families who recently migrated to Canada highlighted a lack of culturally tailored, accessible vaccination information and inadequate cultural competency among HCPs which negatively affected vaccination experiences.^41^ Contrastingly, our study found healthcare providers in East London were attuned to the Bangladeshi community’s needs, thus facilitating uptake.

Generational differences in vaccination decision-making were observed in our study. Among first-generation parents, intrinsic trust and reliance on the advice of HCPs meant the decision to vaccinate was largely one of compliance. This is analogous to previous research with immigrant parents that found parents expressed little agency in their decision to vaccinate their child(ren) and simply followed the HCPs recommendation.^42^ While this inherent trust promotes parental compliance with recommended vaccinations, it is vital to ensure parents, particularly those with lower health and language literacy are supported to make autonomous, informed health decisions through culturally tailored healthcare and decision aids.^43^ Contrastingly, some second and third-generation Bangladeshi parents with higher health literacy expressed more scepticism of vaccines, due to their increased agency, exposure and access to conflicting information online, aligning with previous research.^44^ While this did not translate into vaccine refusal in our study, the growing scepticism suggests the high uptake may be at risk of declining among later generations of the Bangladeshi community if their information needs are not appropriately addressed through needs-driven, targeted educational intervention efforts.^45^

As highlighted in our study, call and recall services have been shown to be effective in improving vaccine uptake,^46 47^ including MMR vaccine uptake in Tower Hamlets.^48^ Bangladeshi parents with lower health literacy expressed a particularly high reliance on these reminders, which were often attuned to their linguistic and informational needs. The tailoring of reminders is an important consideration as general reminders are less effective in improving vaccine uptake for minority groups with literacy and language barriers.^49^ Nevertheless, with increasingly high service demands on GP practices, the sustainability of consistent, tailored call and recall activity may prove challenging.^50^ In a similar regard, the universal provision of a PCHR was recognised as a critical enabler for timely uptake. It is thus key to ensure newly arrived migrants who may experience unique challenges in documenting and tracking vaccinations are informed of and offered this tool at the earliest opportunity to facilitate future uptake.

Finally, the sociocultural context of the Bangladeshi community in East London may be conducive to their positive uptake of childhood vaccinations. The Bangladeshi community, despite being an ethnic minority group nationally, is the largest ethnic group in Tower Hamlets and a settled population.^16^ Accordingly, over time their healthcare utilisation needs have become well understood by service providers, enabling the delivery of needs-tailored healthcare.^2151^,^53^ Large concentrations of an ethnic minority population may experience increased social cohesion and capital, this support and social reinforcement has been recognised to be protective against vaccination hesitancy.^54^ Accordingly, the vaccination enablers identified within this Bangladeshi community may not be entirely transferable to other minority groups, including smaller Bangladeshi communities in other parts of the UK, with less established ties to a location and lower social capital, therefore necessitating targeted research to understand their specific needs.^55^

### Strengths and limitations

A key strength of this study lies in its enquiry with multiple stakeholders. This methodology captured the most important childhood vaccination enablers from the two interconnected perspectives of service users and service providers.

A limitation of our research lies in the representativeness of the parents interviewed. Most parents interviewed were UK-born Bangladeshis who were native English speakers with working knowledge of the vaccination programme and how to access it. Recent migrant Bangladeshi parents and those with an English language barrier, who may experience unique barriers in accessing vaccination services although included, although included were less represented.^56^ Another limitation is that non-immunising parents did not participate in our study despite our broad recruitment efforts. This limits our understanding of the extent to which the enablers we identified drive uptake, and thus should be explored in future research.

### Implications for practice and research

While populations are inherently heterogeneous, our study provides evidence that may inform interventions to improve childhood vaccine uptake among populations with low uptake. From a system-level perspective, it is vital to ensure service providers receive cultural competency training concerning the needs of underserved communities to foster parental trust in HCPs and vaccine recommendations.^57^ However, fostering this trust may require significant structural efforts and targeted research into community dynamics and barriers, particularly for black and minority ethnic groups that have reportedly high levels of distrust in healthcare organisations.^58^

Vaccination providers should maintain a consistent call and recall service, ensuring vaccine reminders are accessible to the populations’ linguistic and informational needs and efficient to administer by primary care staff. The administration of a PCHR may also facilitate the ability of parents to plan, track and attend vaccination appointments.

Beyond system-level factors, this study highlights the importance of using existing trusted social networks to promote vaccination acceptance within a tight-knit community.^59^ Such networks may offer a readily accessible pathway to deliver vaccination messages, particularly for underserved, migrant communities who often demonstrate lower health-seeking behaviour and trust in formal organisations.^60^

## Conclusion

Multi-level enablers to high childhood vaccination uptake were identified for the socioeconomically deprived Bangladeshi community in East London. Trust in the vaccination programme appears central and may be cultivated through the culturally sensitive design of vaccination services, skilled HCPs and the use of trusted social networks to disseminate positive vaccination messages. However, we recognise this community has unique characteristics, such as its long-term settlement and significant presence in Tower Hamlets which mean these enablers may not fully explain differences in uptake across other ethnic minority groups. While the present findings may provide important learnings for communities with suboptimal vaccination uptake, there is no easy nor fast solution to improving uptake, rather concerted investment in long-term, multi-level community engagement initiatives is required.^48^ Future research may explore the feasibility and acceptability of implementing such multi-level engagement efforts to promote vaccine uptake in at-risk communities.

## Supplementary material

10.1136/bmjph-2024-001004 online supplemental material 1

10.1136/bmjph-2024-001004 online supplemental material 2

10.1136/bmjph-2024-001004 online supplemental material 3

## Data Availability

No data are available.
