# Supplementary material for: ‘It was just the given thing to do’: exploring enablers for high childhood vaccination uptake in East London’s Bangladeshi community—a qualitative study
Source: BMJ Public Health. 2025 Jan 16;3(1):e001004. doi: 10.1136/bmjph-2024-001004 (PMC11812862; doi:10.1136/bmjph-2024-001004)
Supplement: online supplemental material 1 [file bmjph-3-1-s001.pdf]

Topic guide for interviews with parents/guardians

Version 1.0; 11.08.2021

Thank you for taking part in this study. I would like to ask you some questions about what you think about vaccinations – focusing on childhood vaccinations, including measles, mumps and rubella - and your experiences of vaccinations.

1. To begin with, perhaps you please tell me a little bit about yourself and your family members?
    - How many children do you have? What are their ages?
    - How long have you lived in Tower Hamlets/Newham?
    - Have you lived in the UK all of your life?

2. Could you tell me [a story] about your experiences of vaccinations? - Perhaps you could tell me about the last time you took your child/your youngest child for a vaccination?

3. Please can you tell me what you think about vaccinations?
4. What have you heard about vaccinations?
5. Have you received vaccinations yourself?
6. Has your child/have your children had all of the vaccinations offered? Why?
7. Do you feel more comfortable with your child receiving some vaccinations than others?

- What vaccinations do you feel more comfortable with? Why?
- What vaccinations do you feel less comfortable with? Why?

8. What are your thoughts about MMR vaccination?
9. What do your family think about vaccinations?
10. What do your friends think about vaccinations?
11. I would now like to ask a little bit about community. Who do you think of as your community? What does your community think about vaccinations?

12. How is the decision made about whether or not to vaccinate your child/children?
    - Who is involved in the decision-making process?
    - Where do you learn about vaccines?
    - What sources of information or do you access?
    - What information sources do you trust most?
13. Where do you take your child/children for vaccination? Can you tell me about your relationship with your GP/practice nurse?
14. Is there anything that makes it easy for your child or children to be vaccinated?
15. Is there anything that makes it hard for your child or children to be vaccinated?
16. Has the COVID-19 pandemic changed your views on vaccinations? If so, how?
17. Is there anything that could be done to make it easier to access vaccinations?
18. Thank you for telling me about your vaccination views and experiences. Is there anything else that you would like to add?
