# Supplementary material for: ‘It was just the given thing to do’: exploring enablers for high childhood vaccination uptake in East London’s Bangladeshi community—a qualitative study
Source: BMJ Public Health. 2025 Jan 16;3(1):e001004. doi: 10.1136/bmjph-2024-001004 (PMC11812862; doi:10.1136/bmjph-2024-001004)
Supplement: online supplemental material 2 [file bmjph-3-1-s002.pdf]

Topic guide for interviews with providers

Version 1.0; 23.08.2021

Thank you for taking part in this study. I would like to ask you some questions about your job role and involvement in working with the Bangladeshi community in Tower Hamlets/Newham. We are particularly interested in your role in relation to childhood vaccines, particularly MMR vaccination.

Job role

1. Firstly, can you please tell me about your job role?
2. How long have you worked in your role?
3. What does your role involve on a day-to-day basis?
4. How are you involved with the Bangladeshi community in Tower Hamlets/Newham?
    - In your role, how frequently do you see Bangladeshi community members?
5. Can you tell me about your role in relation to childhood vaccinations?

Vaccination attitudes

6. How would you describe the attitudes of Bangladeshi community members in Tower Hamlets and/or Newham towards childhood vaccinations? Particularly thinking about MMR vaccination.
7. Does this differ from your experience with other community groups or the population in general?
8. Do attitudes vary across different childhood immunisations? If so, how?
9. Have attitudes changed?

Vaccination uptake

10. What is childhood vaccination uptake like amongst Bangladeshi community members in Tower Hamlets/Newham? Particularly MMR vaccination uptake.
    - Does it differ to uptake amongst other groups in the community?
    - If yes, how does it differ?
    - Why do you think there is a difference?
11. Have you seen any changes in MMR vaccination uptake amongst Bangladeshi community members since the start of the COVID-19 pandemic?
    - If so, what difference have you observed?
    - Why do you think this has happened?

Vaccination delivery

12. Are there any particular approaches used to promote childhood vaccination uptake amongst Bangladeshi communities?
13. Are there particular approaches that you think work well? Please can you describe these.
14. Does your practice/service provide any particular services resources e.g. leaflets/posters, translated documents that specifically aimed at improving childhood vaccination uptake amongst Bangladeshi communities?
15. Do you have any suggestions on how childhood vaccination uptake could be improved amongst Bangladeshi community members?

16. Thank you for telling me about your involvement with Bangladeshi communities and childhood vaccines. Is there anything else that you would like to add?
