# Supplementary material for: ‘It was just the given thing to do’: exploring enablers for high childhood vaccination uptake in East London’s Bangladeshi community—a qualitative study
Source: BMJ Public Health. 2025 Jan 16;3(1):e001004. doi: 10.1136/bmjph-2024-001004 (PMC11812862; doi:10.1136/bmjph-2024-001004)
Supplement: online supplemental material 3 [file bmjph-3-1-s003.pdf]

Supplemental Material 3

Table 2. Demographic characteristics of the Bangladeshi parents interviewed.

**Parent number**	**Age range**	**Migration status of parent**	**Self-reported immunisation status of child(ren)**
1	30-39	Born in the UK	Received all recommended vaccinations to date.
2	20-29	3 years in the UK	Received all recommended vaccinations to date.
3	30-39	Lived in the UK since age of 6 months	Received all recommended vaccinations to date.
4	30-39	Lived in the UK since age of 4 years	Received all recommended vaccinations to date.
5	30-39	4 years in the UK	Received all recommended vaccinations to date.
6	30-39	10 years in the UK	Received all recommended vaccinations to date.
7	Not disclosed	Born in the UK	Received all recommended vaccinations to date.
8	20-29	Born in the UK	Received all recommended vaccinations to date.
9	Not disclosed	15 years in the UK	Received all recommended vaccinations to date.
10	30-39	10 years in the UK.	Received all recommended vaccinations to date.

11	30-39	13 years in the UK	Received all recommended vaccinations to date.
12	20-29	Not disclosed	Received all recommended vaccinations to date.
13	30-39	Born in the UK	Received all recommended vaccinations to date.
14	30-39	Born in the UK	Received all recommended vaccinations **except Flu vaccine**.
15	40-49	Born in the UK	Received all recommended vaccinations to date.
16	40-49	20 years in the UK	Received all recommended vaccinations to date.
17	30-39	10 years in the UK	Received all recommended vaccinations to date.
18	40-49	7 years in the UK	Received all recommended vaccinations to date.
19	30-39	15 years in the UK	Received all recommended vaccinations to date.
20	30-39	5 years in the UK	Received all recommended vaccinations to date.
21	Not disclosed	Born in the UK	Unable to recall.
22	Not disclosed	Born in the UK	Received all recommended vaccinations to date.
23	30-39	Born in the UK	Received all recommended vaccinations to date.
